# Single-Port versus Multiple-Port Robot-Assisted Radical Prostatectomy: A Systematic Review and Meta-Analysis

**DOI:** 10.3390/jcm10245723

**Published:** 2021-12-07

**Authors:** Omar Fahmy, Usama A. Fahmy, Nabil A. Alhakamy, Mohd Ghani Khairul-Asri

**Affiliations:** 1 Department of Urology, Universiti Putra Malaysia (UPM), Serdang 43400, Selangor, Malaysia; omarfahmy.ahmed@upm.edu.my; 2 Department of Pharmaceutics & Industrial Pharmacy, Faculty of Pharmacy, King Abdulaziz University, Jeddah 21589, Saudi Arabia; uahmedkauedu.sa@kau.edu.sa (U.A.F.); nalhakamy@kau.edu.sa (N.A.A.); 3 Center of Excellence for Drug Research and Pharmaceutical Industries, King Abdulaziz University, Jeddah 21589, Saudi Arabia; 4 Mohamed Saeed Tamer Chair for Pharmaceutical Industries, King Abdulaziz University, Jeddah 21589, Saudi Arabia

**Keywords:** prostate cancer, radical prostatectomy, robotic prostatectomy, single port, laparoscopy

## Abstract

Background: Single-port robotic-assisted radical prostatectomy has been reported as a safe and feasible technique. However, recent studies comparing single-port versus multiple-port robotic radical prostatectomy have displayed conflicting results. Objectives: To investigate the benefit of single-port robotic radical prostatectomy and the impact on outcome compared to multiple-port robotic radical prostatectomy. Methods: Based on PRISMA and AMSTAR criteria, a systematic review and meta-analysis were carried out. Finally, we considered the controlled studies with two cohorts (one cohort for single-port RARP and the other cohort for multiple-port RARP). For statistical analysis, Review Manager (RevMan) software version 5.4 was used. The Newcastle-Ottawa Scale was employed to assess the risk of bias. Results: Five non-randomized controlled studies with 666 patients were included. Single-port robotic radical prostatectomy was associated with shorter hospital stays. Only 60.6% of single-port patients (109/180) required analgesia compared to 90% (224/249) of multiple-port patients (Z = 3.50; *p* = 0.0005; 95% CI 0.07:0.47). Opioid administration was also significantly lower in single-port patients, 26.2% (34/130) vs. 56.6% (77/136) (Z = 4.90; *p* < 0.00001; 95% CI 0.15:–0.44) There was no significant difference in operative time, blood loss, complication rate, positive surgical margin rate, or continence at day 90. Conclusion: The available data on single-port robotic radical prostatectomy is very limited. However, it seems comparable to the multiple-port platform in terms of short-term outcomes when performed with expert surgeons. Single-port prostatectomies might provide a shorter hospital stay and a lower requirement for opioids; however, randomized trials with long-term follow-up are mandatory for valid comparisons.

## 1. Introduction

Prostate cancer is the second most common cancer diagnosis in males and the fifth-greatest cause of death globally. In 2018, almost 1.3 million new instances of prostate cancer were recorded worldwide, with developed countries having a greater frequency [1]. Radical prostatectomy is one of the primary therapeutic choices for prostate cancer, including locally advanced disease [2], and approximately half of all prostate cancer patients will have radical prostatectomy [3]. Robotic-assisted laparoscopy is now used in more than 80% of radical prostatectomy procedures due to lower complications and earlier recovery as compared to the open method [4].

Multi-port (MP) da Vinci^®^ Xi and Si (Intuitive Surgical, Sunnyvale, CA, USA) are now the most widely utilized robotic systems in urologic procedures [5,6]. With further advancements in technology, the single port (SP) da Vinci system was introduced a few years ago for further minimization of surgical invasiveness; it has been approved by the Food and Drug Administration (FDA) since 2018. Several urologic operations performed with the SP robotic system have been documented, with radical prostatectomy being the most frequent [7].

Theoretically, the SP platform needs a single incision at the midline, which carries the advantage of avoidance of the other lateral incisions required for the MP platform. This could reduce postoperative pain and possible surgical wound complications. In addition, the flexibility of the camera and instruments due to the “two points of articulation” helps the surgeons to operate in a smaller surgical field and identify the ideal position for the camera and instruments for each surgical step [7].

The feasibility and safety of SP RARP have been demonstrated in early publications [8]. A few studies comparing the intraoperative performance and early functional and oncological results of these two robotic systems (SP vs. MP) have recently been published. However, the advantages of SP have shown contradictory findings. Some studies have linked lower postoperative pain and shorter hospital stays to SP [9,10], whereas others have shown no difference between SP and MP in terms of postoperative pain [11]. Surprisingly, some studies found reduced blood loss and a lower rate of positive surgical margins associated with SP [9] in contrast to other studies [10].

The aim of this work is to investigate the benefits of the SP da Vinci system in RARP and to measure its perioperative, functional, and oncologic results against the MP system.

## 2. Materials and Methods

### 2.1. Search Strategy

According to PRISMA and AMSTAR criteria [12,13], an online systemic search was conducted through online databases (PubMed, EMBASE, Wiley Online Library, and Cochrane databases). The following keywords were utilized; robotic prostatectomy; robotic-assisted prostatectomy; radical prostatectomy; prostate cancer; single-port. The time frame of the search was from January 2000 until November 2021. The inclusion criteria were: (1) English publications; (2) controlled studies, either randomized or non-randomized, with one arm for single-port and the other arm for multiple-port robotic radical prostatectomy; (3) prospective or retrospective studies. The exclusion criteria were: (1) review articles; (2) case reports; (3) letters to editors and editorial comments; (4) repeated publications from the same author or from the same center; (5) case series containing one kind of intervention (no control arm); (6) non-English articles. All initial results underwent staged selection and screening, the first stage by assessing the title and abstract to exclude unrelated articles, review articles, editorial comments, and case reports and the second stage by full-text assessment to exclude repeated publications and non-controlled case series. A manual search was performed in the reference lists of the selected papers and the excluded review articles to avoid missing any eligible publications.

### 2.2. Data Extraction

The following variables were independently extracted by two authors and checked by a third one: total number of patients, age, operative time (OT) in minutes, estimated blood loss (EBL) in mL, length of hospital stay (LHS) in days, pain score, patients who required analgesia, pain-free patients (who did not require postoperative analgesia), complications, positive surgical margins (PSMs), number of continent patients at day 90. Any discrepancy in the extracted data was dissolved after discussion among all the authors. All the variables were reported and extracted in numbers or mean and standard of deviation (SD). When data were reported as median and range or interquartile range, Wan’s equation was applied to estimate the mean and SD from the median, range/interquartile range, and sample size [14].

### 2.3. Primary Outcomes

The primary outcome of this systematic review and meta-analysis was to compare SP vs. MP RARP in terms of perioperative, oncological, and functional outcomes. Based on the extracted data, perioperative outcomes were assessed by EBL, OT, LHS, pain score, requirement for opioids, and complications. Oncological and functional outcomes were assessed by PSM and continence at day 90, respectively.

### 2.4. Statistical Analysis

The Nordic Cochrane Centre, The Cochrane Collaboration, Copenhagen, employed Review Manager (RevMan) software version 5.4 for statistical analysis and the creation of forest plots for this meta-analysis. The mean difference with the 95% confidence interval (CI) was utilized for continuous data (EBL, OT, LHS, pain score). For dichotomous data (complications, pain-free, opioid requirement, PSM, continence at day 90), we utilized the odds ratio (OR) with 95% CI. The I2 value was used to determine the heterogeneity of the research. For I2 < 50%, the random effect model was utilized in all the analyses regardless of the I^2^ value to minimize the effect of heterogeneity among the studies. The Z-test was used to assess the overall impact. *p*-values < 0.05 were deemed significant in all tests.

### 2.5. Risk of Bias Assessment

The Newcastle-Ottawa Scale (NOS) was employed in this meta-analysis to assess the quality of non-randomized trials [15]. Scores of 7–9, 4–6, and 4 were classified as having a low, moderate, or high risk of bias, respectively.

## 3. Results

### 3.1. Search Results

**Figure 1:**
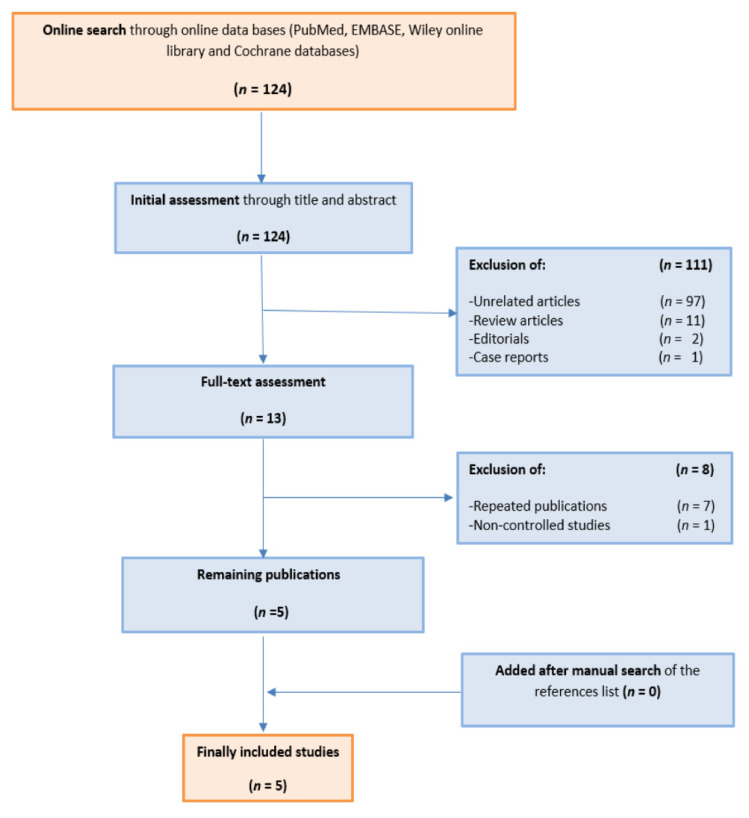
PRISMA diagram for the flow of selection process of the included studies.

The initial search in the electronic databases displayed 124 articles, which underwent initial assessment followed by the exclusion of 111 results. A further eight results were excluded in the second stage of the full-text assessment. Eventually, five non-randomized studies with 666 patients were included [9,10,11,16,17]. Of the total of 666 patients, 298 (44.7%) underwent SP-RARP and 368 (55.3%) underwent MP-RARP. Table 1 provides an overview of the included studies. A NOS risk of bias evaluation revealed that all studies are within the low-risk category (Table 2). All the included studies failed to gain a star for adequate follow-up duration except for Lenfant et al.’s study, which had a follow-up of more than 12 months [16]. Three of five studies lost one star for comparability due to some heterogeneity between the experimental and control arms in each study [11,16,17]. The flow of the screening and selection processes is demonstrated in the PRISMA diagram in Figure 1.

### 3.2. Perioperative Outcomes

**Figure 2:**
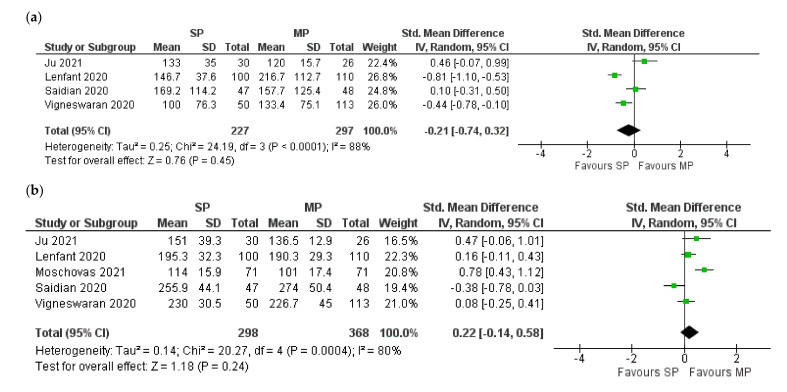

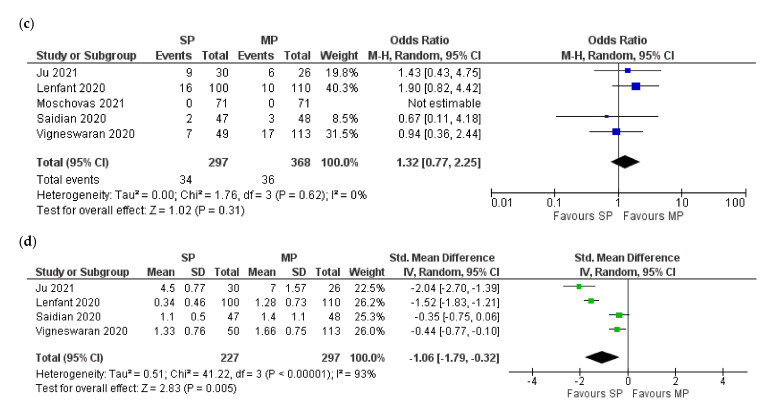
Forest plots for the comparison of the perioperative outcomes: (**a**) estimated blood loss; (**b**) operative time; (**c**) complications; (**d**) length of hospital stay. SP: single port; MP: multiple-port.

In total, 4 studies with 524 patients compared the EBL [9,10,16,17]. Overall, there was no significant difference between SP and MP RARP (Z = 0.76; *p* = 0.45; 95% CI −0.74:0.32). (Figure 2a). For OT and complication rate, all the five studies were included. Additionally, no significant differences were noted: Z = 1.18; *p* = 0.24; 95% CI −0.14:0.58 and Z = 1.02; *p* = 0.31; 95% CI 0.77:2.25 for OT and complications, respectively (Figure 2b,c).

LHS was significantly shorter in the SP group based on the pooled analysis of four studies with 524 patients [9,10,16,17] (Z = 2.83; *p* = 0.005; 95% CI −1.79:−0.32) (Figure 2d).

**Figure 3:**
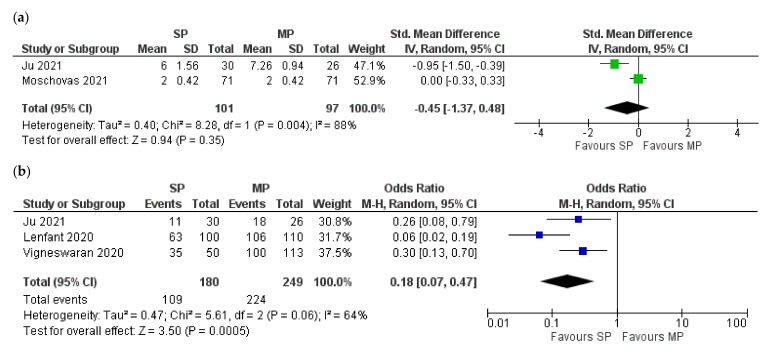

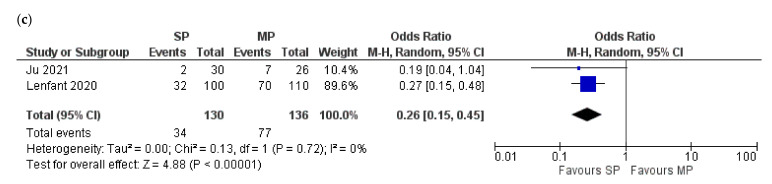
Forest plots for the comparison of postoperative pain: (**a**) pain score; (**b**) pain-free patients; (**c**) requirement for opioids. SP: single port; MP: multiple-port.

Based on the available data, postoperative pain was compared using pain score, number of pain-free patients, and requirement for opioids. From the combined analysis of two studies with 198 patients [10,11], the difference in pain score was non-significant (Z = 0.94; *p* = 0.35; 95% CI −1.37:0.48). In the pooled analysis of three studies [9,10,16], only 60.6% of SP patients (109/180) required analgesia compared to 90% (224/249) of MP patients (Z = 3.50; *p* = 0.0005; 95% CI 0.07:0.47). Opioid administration was also significantly lower in SP patients: 26.2% (34/130) vs. 56.6% (77/136) [9,10], (Z = 4.90; *p* < 0.00001; 95% CI 0.15: −0.44) (Figure 3).

### 3.3. Oncological Outcomes

**Figure 4:**
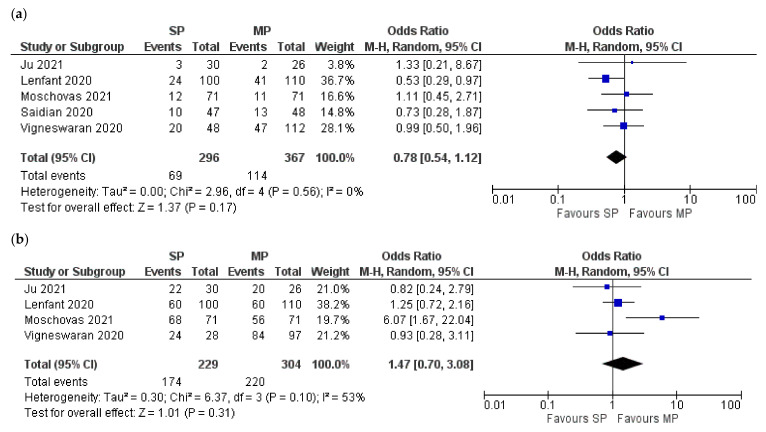
Forest plots for the comparison of positive surgical margins (**a**) and continence at day 90 (**b**). SP: single port, MP: multiple-port).

Pooled analysis for PSM was the only feasible comparison to assess the oncological outcomes. SP displayed a trend toward a lower percentage of PSM but could not reach a significant level. It was 23.3% (69/296) for SP vs. 31% (114/367) for MP [9,10,11,16,17] (Z = 1.39; *p* = 0.16; 95% CI 0.54:1.11) (Figure 4a).

### 3.4. Functional Outcomes

Continence at day 90 was the only possible variable to be involved in a meta-analysis of four studies for functional outcome [9,10,11,16]; 76% (174/229) of SP patients were continent at day 90, compared to 72.4% (220/304) of MP patients (Z = 1.01; *p* = 0.31; 95% CI 0.70:3.08) (Figure 4b).

## 4. Discussion

Prostate cancer is the world’s second most frequent malignant tumor in males. With increased life expectancy, more patients diagnosed with prostate cancer might receive surgical treatment [18]. William Schuessler performed the first laparoscopic-assisted radical prostatectomy in Texas in 1991 to reduce the morbidity of open surgery and overcome the challenging exposure of the retropubic region during open radical prostatectomy [19]. Binder in Frankfurt, Germany, and Abbou in Creteil, France, performed the first RARP in 2000 [20,21]. Over time, it grew in popularity, and, currently, robotic assistance is used in 80–85% of radical prostatectomies performed in the United States; however, this proportion is lower in Europe [4,22].

The da Vinci SP system is different from MP systems in terms of docking the robot and the types of instruments available. The experience of the assistant surgeon is more important in the SP system compared to the MP system due to the limited ability of retraction of nearby organs, which adds more technical challenges [23]. However, the SP platform could have theoretical advantages over the MP one: SP RARP has better cosmetic outcomes due to less scaring, which could be important for some patients [24]. In a recent report by Noel et al., when the number of incision sites was assessed using the validated Surgical Satisfaction Questionnaire (SSQ-8), patients who had MP had lower satisfaction percentages than those who received SP. These patients attributed their dissatisfaction to the number and look of scars [25]. In addition, it limits the need for adhesiolysis as the peritoneum cavity is entered at only one point. Due to the lack of a lateral port, adhesions in the lateral parts of the abdomen, which are commonly seen as a result of appendicitis or diverticulitis, do not need to be treated [26]. Furthermore, direct access to the prostate with minimum peritoneal cavity manipulation can help to avoid postoperative ileus and hasten recovery [27].

One important point when comparing a conventional surgical technique and a novel one is the impact of the learning curve. In the case of shifting to SP after MP RARP, surgical experience can be obtained quickly for surgeons who are already experts in using the MP platform. Even different approaches for RARP have been reported to be feasible by the SP platform. For example, Retzius-sparing and trans-vesical RARP are also challenging with the MP system [28]. Previous experience with single-port laparoscopic surgery might help to shorten the learning curve for the SP robotic platform. Additionally, training sessions on cadavers or animals are useful before performing real surgeries on patients [27]. However, all the studies included in our analysis are from high-volume centers, where conventional robotic radical prostatectomy with the multiple-port platform is well established, and surgical teams have completed their learning curve. Therefore, fewer difficulties would be anticipated with their shift to the single-port platform. For surgeons without enough experience with conventional robotic radical prostatectomy, single-port surgery can be the next step only after mastering the conventional technique.

In our results, there was no significant difference in operative time. In our opinion, this should mean that SP RARP is not challenging because the main challenging part in RARP is the console surgery. However, in SP RARP, some steps are expected to save time compared to the MP approach, such as insertion of the port and docking at the start and port site closure at the end. Furthermore, there are intraoperative steps, such as nerve-sparing, pelvic lymphadenectomy, and bladder neck reconstruction, that can affect operative time and are not performed for every patient.

Fundamental limitations must be taken into consideration when interpreting the results of this systematic review. Mainly, it is based on a few retrospective studies with a small number of patients and short-term follow-up. The absence of randomization increases the risk bias of patient selection, especially when starting a new surgical technique, to avoid any difficulty. In addition, we lack answers to important questions such as proper cost-analysis, biochemical recurrence, and erectile function after RARP. Despite the NOS assessment of bias displaying a low risk of bias, many forest plots showed moderate and high degrees of statistical heterogeneity. This could be due to the marked diversity in the reported outcomes among the studies.

It is usually acceptable for initial reports on new surgical techniques to be retrospective as it is infeasible to run a randomized trial without enough evidence on the safety and efficacy of the tested approach. For future studies, ideally, they should consider comparing not only the long-term outcomes but also the financial aspects of shorter hospital stays and less painkiller administration in addition to any differences in robotic system purchasing, maintenance, and running costs in the long term.

## 5. Conclusions

The available data on single-port robotic radical prostatectomy is very limited. Based on the published preliminary results, it seems comparable to the multiple-port platform in terms of safety and short-term outcomes, with shorter hospital stays, less postoperative pain, and a lower requirement for opioids. Single-port radical prostatectomy can be considered a future step for only expert surgeons who have already completed their learning curve for multiple-port robotic prostatectomy. Randomized trials with long-term follow-up are necessary for valid investigation of the real benefits of the single-port system.

## Figures and Tables

**Table 1:** Characteristics of the included studies.

Study	Time Frame	Country	Sample Size		Median (Range) Follow-Up Months	
			SP	MP	SP	MP
Moschovas 2021 [9]	June 2019–April 2020	USA	71	71	4.4 (1.6–7.2)	3.2 (1.6–4.8)
Ju 2021 [10]	April 2019–March2020	China	30	26	3	3
Saidian 2020 [11]	October 2018–June 2019	USA	47	48	NA	NA
Lenfant 2020 [16]	January 2019–January 2020	USA	100	110	12	12
Vigneswaran 2020 [17]	Sepember 2017–November 2019	USA	50	113	4.7 (4.3–4.3)	7.7 (5.0–12.7)

SP: single port; MP: multiple port; NA: not available.

**Table 2:** Newcastle-Ottawa Scale for risk of bias assessment of the included studies (scores ≥ 7–9, 4–6, <4 are considered low, intermediate, and high risk, respectively).

Study	Selection				Comparability	Outcome			Overall
	Representativeness of Exposed Cohort	Selection of Non-Exposed	Ascertainment of Exposure	Outcome Not Present at Start		Assessment of Outcome	Adequate Follow-Up Length	Adequacy of Follow-Up	
Moschovas 2021 [9]	*	*	*	*	**	*	-	*	8/9
Ju 2021 [10]	*	*	*	*	**	*	-	*	8/9
Saidian 2020 [11]	*	*	*	*	*-	*	-	*	7/9
Lenfant 2020 [16]	*	*	*	*	*-	*	*	*	8/9
Vigneswaran 2020 [17]	*	*	*	*	*-	*	-	*	7/9

* Equals one point, with a total of 9 points (* for each item and ** equals 2 points for comparability).

## Data Availability

Not applicable.
